# Health-Related Quality of Life in Patients with Inflammatory Bowel Disease in Clinical Remission: What Should We Look For?

**DOI:** 10.3390/medicina58040486

**Published:** 2022-03-27

**Authors:** Tudor Stroie, Carmen Preda, Corina Meianu, Adina Croitoru, Liana Gheorghe, Cristian Gheorghe, Mircea Diculescu

**Affiliations:** 1 Faculty of Medicine, “Carol Davila” University of Medicine and Pharmacy, 050474 Bucharest, Romania; preda_monicaa@yahoo.com (C.P.); corina_meianu@yahoo.com (C.M.); liliana.gheorghe@umfcd.ro (L.G.); cristian.gheorghe@umfcd.ro (C.G.); mihai.diculescu@umfcd.ro (M.D.); 2 Department of Gastroenterology, Fundeni Clinical Institute, 022328 Bucharest, Romania; 3 Faculty of Medicine, “Titu Maiorescu” University, 031593 Bucharest, Romania; adina.croitoru09@yahoo.com; 4 Department of Oncology, Fundeni Clinical Institute, 022328 Bucharest, Romania

**Keywords:** IBD, quality of life, anxiety, depression, fatigue

## Abstract

*Background and Objectives*: Inflammatory bowel diseases (IBD) are chronic conditions with an unpredictable evolution that can have a negative impact on patients’ quality of life (QoL). Even though patients in remission have a better QoL compared to patients with active disease, they still have a lower QoL compared to healthy people. The aim of this study is to identify the factors that are associated with a lower QoL in patients with IBD in clinical remission, in a tertiary IBD center in Romania. *Materials and Methods*: Ninety-seven adult patients with a current diagnosis of IBD for over 3 months who were in clinical remission were enrolled in this study. Pregnant women, patients with ostomy, perianal disease, extraintestinal manifestations or other significant comorbidities were excluded. Out of the 97 patients, 63.9% were men. The median age was 39 years (IQR 29–47), and the median disease duration was 5 years (IQR 2–10). Disease activity was assessed using the SCCAI score for ulcerative colitis and HBI score for Crohn’s disease. Remission was defined for SCCAI score ≤ 1 and HBI score ≤ 4. The health-related quality of life (HR-QoL) was assessed using the IBDQ32 score. FACIT-Fatigue was used to evaluate the level of fatigue. Patients with symptoms of anxiety or depression were identified with the HADS score. Symptoms of anxiety were considered when HADS-A >7 points and symptoms of depression when HADS-D >7 points. *Results*: Sixty-five patients (67%) were diagnosed with CD and the remaining 32 (33%) with UC. Ninety-three patients (95.9%) were on biological therapy. The mean IBDQ score (total score) was 190.54 points (SD +/− 8.2). The mean FACIT Fatigue score was 42.5 (SD +/− 8.2), with 6.2% of patients suffering from severe fatigue (FACIT Fatigue < 30 points). A total of 33% of patients had symptoms of anxiety and 16.5% of depression. Exposure to more than one biologic therapy (*p* = 0.02), fatigue (*p* < 0.001) and symptoms of anxiety (*p* < 0.001) were associated with a lower HR-QoL in the multivariate analysis. Female patients, patients with Crohn’s disease, patients with anemia and patients with symptoms of depression also had a lower HR-QoL, but this did not reach statistical significance in our study. *Conclusions*: Exposure to a higher number of biological agents (patients that switched multiple biologics), the presence of fatigue and symptoms of anxiety impair the HR-QoL of patients with IBD in clinical remission. Further studies should assess in a prospective manner whether early identification of these factors with prompt clinical interventions could lead to a better HR-QoL in these patients.

## 1. Introduction

Inflammatory bowel diseases (IBD) represent chronic conditions with a remitting relapsing course, which affect mainly young patients and with a clinical evolution that can be often debilitating. There are two subtypes of IBD: Crohn’s disease (CD) and ulcerative colitis (UC). Their etiologies are multifactorial and incompletely elucidated, but factors like genetic predisposition, altered immune response, complex interactions with environmental factors or alteration in the intestinal microbiome can be involved in the etiology [1]. The prevalence of IBD is increasing worldwide, with prevalences up to 322/100,000 inhabitants for CD and 505/100,000 inhabitants for UC in Europe [2].

Because of its chronic nature and unpredictable course, symptoms like abdominal pain, chronic diarrhea, urgency, abdominal pain or weight loss can play a significant role in impacting patients’ quality of life (QoL), affecting many aspects of their daily lives. Moreover, the permanent fear of requiring surgical interventions, the distress around ending up with an ostomy, or the concerns around the increased risk of developing cancer may further impair their QoL [3].

Over the last few decades, the study of QoL has received increasing interest from the medical research community, with a higher number of studies being published every year. Although neglected in the past, health-related quality of life (HR-QoL) has become one of the major endpoints of clinical studies [3].

Patients with IBD have a lower HR-QoL compared to healthy individuals or the general population, with lower QoL scores in both the mental and physical domains. Among patients with IBD, those with active disease have a significantly lower QoL compared to patients in remission [3,4].

There is a controversy regarding the influence the disease phenotype has in determining the QoL. Even though patients with CD tend to have a lower QoL compared to patients with UC when the disease is in remission, the results reported by clinical studies are divergent; however, in a comprehensive metanalysis, the differences were only borderline significant in the pooled estimate for the mental QoL scores, but they were not significant with generic or IBD-specific QoL scores. Patients with active disease seem to have the same degree of impairment in the QoL, irrespective of the disease phenotype [3,4].

Fatigue is a common and debilitating condition, frequently encountered in patients with IBD, with a multifactorial etiology in which disease activity and nutritional deficiencies play an important role, with studies reporting prevalences up to 41% in patients with IBD in remission. Physical and mental exhaustion, lack of appetite, and demotivation are common complaints among patients with IBD, especially in those with active disease, but also, though in a lesser degree, in patients in remission [5,6,7]. Chronic fatigue has a negative impact on the HR-QoL and is associated with a significant reduction in the QoL, strong correlations being reported between fatigue and HR-QoL scores in patients with IBD [8].

A significant number of patients with IBD are affected by anxiety and depression, which can further impair their QoL. Symptoms of anxiety and depression are reported in up to 35.1% and 21.6% of patients with IBD. Even if depression is reported to be more frequent in patients with CD than in UC patients (25.3% vs. 16.7%), there are no significant differences between the disease phenotypes in regard to anxiety. Of note, both anxiety and depression are more frequent in patients with active disease than in those in remission (75.6% vs. 31.4% for anxiety and 40.7% vs. 16.5% for depression) [9].

Patient reported outcome (PRO) measures are standardized, validated questionnaires that are completed by patients, and evaluate their subjective perception over certain aspects such as the quality of life, the functional status or general well-being among many others [10]. A PRO measurement tool can evaluate a single domain (e.g., pain) or several domains (e.g., social function, emotional function, aspects regarding the treatment or the disease). The use of PROs can help clinicians not only by guiding and improving medical care and centering in on the patient, but also by evaluating the patient’s needs or by assessing the different types of medical practices [11].

IBDQ 32 (Inflammatory Bowel Disease Questionnaire) is one of the most widely used questionnaires to evaluate the HR-QoL in patients with IBD. It was developed by Guyatt et al. in 1989 in order to provide a more comprehensive insight of the disease, including its emotional and social aspects. It consists of 32 questions that evaluate 4 domains: bowel symptoms (IBDQ B), systemic symptoms (IBDQ S), emotional function (IBDQ E) and social function (IBDQ SF). Each question is scored from 0 to 7 points, with a total score ranging from 0 to 224, higher scores representing a better HR-QoL [12].

FACIT-Fatigue (the Functional Assessment of Chronic Illness Therapy–Fatigue) is a 13 items questionnaire that measures the degree of general fatigue in patients affected by chronic illnesses, including IBD. It is a subscale of the FACIT questionnaire [13]. Each item is scored between 0–4 points. The scores for all items are summed up to a final score that ranges from 0 to 52, higher scores representing less fatigue [14].

HADS (Hospital Anxiety and Depression Scale) is a 14 items questionnaire. Its purpose is to screen patients for clinically significant anxiety and depression (7 items evaluating each of them). A score over 7 is considered pathological for both anxiety and depression as follows: 8–10 mild anxiety/depression, 11–14 moderate anxiety/depression and >14 severe anxiety/depression [15,16].

## 2. Materials and Methods

### 2.1. Study Population

One-hundred-and-ten consecutive patients with a current diagnosis of IBD for over 3 months who were in clinical remission were invited to participate in this study. Out of this group, 103 patients expressed their consent to participate. Six patients were excluded due to incomplete data and 97 patients were further included in the analysis.

Out of the 97 patients, 63.9% were men. The median age was 39 years (IQR 29–47), and the median disease duration was 5 years (IQR 2–10). A total of 67% of patients were diagnosed with CD and the rest of 32 (33%) with UC. 61.9% of them were active smokers (Table 1).

The patients were enrolled in the study during a routine follow-up visit. Pregnant women and patients with ostomy, perianal disease, extraintestinal manifestations, or other significant comorbidities that could interact with their quality of life were excluded. All patients were at least 18 years old and expressed their consent to participate in this study.

### 2.2. Questionnaires and Data Collection

Patients were invited to complete 3 questionnaires to evaluate their HR-QoL, fatigue, and the presence of symptoms of anxiety and depression: IBDQ32, FACIT-Fatigue and HADS. Symptoms of anxiety were considered when HADS-A >7 and symptoms of depression when HADS-D >7.

The blood samples were collected before the patients’ routine follow-up visit at the hospital and after the completion of the questionnaires. Complete blood count and the CRP level were collected for all patients included in this study. The level of CRP was used to certify the remission and the hemoglobin level to assess for anemia. Anemia was considered as hemoglobin level <13 g/dL in men and <12 g/dL in women.

Demographic data and data regarding the disease were collected from the patients after completing the questionnaires and from the informatic database of the hospital.

### 2.3. Assessment of Disease Activity

Clinical remission was assessed using the Harvey Bradshaw Index (HBI) for Crohn’s disease and Simple Clinical Colitis Activity Index (SCCAI) for ulcerative colitis. Remission was defined when HBI ≤4 points and SCCAI ≤ 1 points. All the patients included had a normal CRP level in order to ascertain remission.

### 2.4. Statistical Analysis

Statistical analysis was performed using R version 4.1.2.

A sample size of 90–110 patients was needed to allow the use of a multivariate linear regression with 9–11 covariates and to avoid sparse data problems.

Firstly, a bivariate analysis was done using parametric tests (*t* test and ANOVA) to compare the means of continuous variables represented by the IBDQ score between different categories of patients stratified by gender, phenotype, number of biological therapies, level of education, presence of anemia and presence of symptoms of anxiety or depression.

Pearson correlation was used to assess the level of correlation between IBDQ32 score and age, disease duration, and FACIT Fatigue score.

Secondly, a multiple linear regression model was used for the multivariate analysis, including the variables analyzed in the bivariate analysis. A subanalysis of IBDQ32 score’s domains was performed.

The statistical significance was considered as *p* < 0.05.

### 2.5. Ethics

The study was approved by the local ethics committee of Fundeni Clinical Institute.

All patients were informed about the details and the purpose of this study, including information on the methodology and the risk and benefits of participating in this study. They were assured about the confidentiality of their personal data. All the patients that agreed to participate signed an informed consent form before any other study procedure was done: the completion of the questionnaires and the collection of personal data (demographics, data regarding the disease and the treatment and results from blood tests).

## 3. Results

Out of the 110 patients that were invited to participate in this study, 103 expressed their agreement to be enrolled and signed the informed consent form, and 97 patients had complete data and could be included in this study (Table 1).

Regarding the treatment, 95.9% were on biological therapy, the majority of them with infliximab (56.7%). A smaller number of patients (10.3%) were treated with adalimumab, 26.8% with vedolizumab and only two (2%) were treated with ustekinumab. There were no patients on corticotherapy, either oral or intravenous. The exposure to previous biological treatments was taken into consideration: 77.3% of patients were exposed to a maximum of one biological treatment and 33.7% were exposed to more than one biologic (they switched multiple biologics). A total of 31.9% of patients had a history of surgical interventions for IBD.

The level of education was assessed and defined as inferior for patients that had only completed the primary cycle of education (6.2%), medium for those who completed high school studies (44.3%) and superior for those that graduated from university (49.5%).

Anemia was present in 10.3% of patients.

The mean IBDQ score (total score) was 190.54 points (SD +/− 8.2). The mean FACIT Fatigue score was 42.25 (SD +/− 8.2), with 6.2% of patients suffering from severe fatigue (FACIT Fatigue < 30 points). Thirty-three percent of patients had symptoms of anxiety and 16.5% of depression.

### 3.1. Bivariate Analysis

The health-related quality of life assessed with the IBDQ 32 questionnaire was compared between different categories of patients, in order to identify factors associated with a lower HR-QoL (Table 2).

Female patients were shown to have a lower HR-QoL compared to men (IBDQ32 Δ = 12.2, *p* = 0.016). Patients with CD had a significantly lower HR-QoL compared to patients with UC (IBDQ32 Δ = 10.91, *p* = 0.007). Regarding the exposure to biological therapy, patients exposed to more than one biological therapy had a significantly poorer HR-QoL compared to those exposed to a maximum of one biologic agent (IBDQ32 Δ = 25.75, *p* = 0.039). Anemia also appears to be a factor associated with a lower HR-QoL (IBDQ32 Δ = 23.25, *p* = 0.02). Significant correlation was noticed between the IBDQ32 score and the FACIT Fatigue score, with r = 0.89 and *p* < 0.001, meaning that fatigue is strongly associated with a lower HR-QoL. The presence of symptoms of anxiety and depression significantly impaired the HR-QoL of patients (IBDQ32 Δ = 35.23, *p* < 0.001 for anxiety and Δ = 33.14, *p* < 0.001 for depression).

Regarding the disease duration, there was a statistically significant yet weak invers correlation with the HR-QoL, with an r = −0.24, *p* = 0.017, which was not considered of clinical significance.

Other factors like age, history of IBD surgery or level of education were not associated with lower HR-QoL.

### 3.2. Multivariate Analysis

All factors analyzed in the bivariate analysis were included in a multiple linear regression model, with the aim to better isolate the factors that are associated with a lower HR-QoL. (Table 2).

Three factors associated with a lower HR-QoL remained statistically significant in the multivariate analysis: exposure to more than one biological agent (*p* = 0.02), fatigue (*p* < 0.001) and anxiety (*p* < 0.001).

All other factors, such as gender, CD phenotype, disease duration, anemia or the symptoms of depression, that were significant in the bivariate analysis, were not statistically significant.

The subanalysis of the factors that lower the QoL for each domain of the IBDQ32 (Table 3) reveals that lower scores were registered in all domains in patients with lower FACIT Fatigue scores (lower QoL correlates with higher level of fatigue). Fatigue was the only factor that lowered the bowel symptoms domain. In addition to fatigue, the systemic symptoms domain had lower scores in patients with younger age and symptoms of anxiety; the emotional function was impaired in patients of younger age, patients with anemia, and patients with symptoms of anxiety. The social function was the only domain that was highly influenced by the number of biological agents the patient was exposed to.

The type of biological therapy did not have a significant impact on the patients’ HR-QoL. The highest HR-QoL was observed among patients treated with infliximab (mean IBDQ score: 195.67); patients treated with adalimumab and vedolizumab had a mean IBDQ score of 185.07 and 185.07, respectively. The lowest HR-QoL was noticed for patients treated with ustekinumab, but there were only two patients in this category. These results were borderline significant in the bivariate analysis (*p* = 0.05) and non-significant when included in a multivariate regression model (*p* = 0.27).

## 4. Discussion

To our knowledge, this is the first study to assess the HR-QoL in patients with IBD in clinical remission in Romania. Patients with IBD in Romania present particular interest due to the fact that data coming from the Eastern Europe regarding their QoL are scarce, with only few articles published so far. Thus, this study presents new insights regarding the factors that impact the HR-QoL of patients with IBD in clinical remission from an understudied population of patients.

Demonstrated by multiple studies, even though patients in remission have significantly better QoL compared to patients with active disease [4], they still have a lower QoL compared to the general population [17] and there are factors that could adversely impact the QoL of these patients as well.

Exposure to more biological treatments was associated with a lower QoL, and this may be due to the fact that patients that needed to switch several times their treatment may have had a more severe disease with a protracted course, more relapses, more intestinal damage, more hospitalizations. This may have impaired their QoL, even though these aspects were not analyzed in this study. They also may be more concerned about their disease, about the lack of further treatments or about the possibility of requiring a surgical intervention.

However, disease duration was not associated with a lower QoL, which may be explained by the fact that there is an adjustment over time to the chronic condition with patients improving their coping mechanisms [18]. In addition, even though cross-sectional studies fail to capture a significant correlation between disease duration and QoL, several longitudinal studies have shown an improvement of the HR-QoL due to this process [4,19]. The same can be said about surgical interventions; in our study, the history of having an IBD-related surgical intervention did not play a role in determining the patient’s HR-QoL, neither in the bivariate nor in the multivariate analysis. Conversely, other studies report significantly lower HR-QoL in patients with both CD or UC requiring surgery at diagnosis or during follow-up, with a significant improvement after the surgical intervention [19,20]. It has also been reported that patients with previous surgery have a better food-related QoL [21].

In our study, the phenotype did not play an important role in impairing the QoL of patients with IBD in clinical remission. Even though patients with CD had a lower IBDQ32 score compared to patients with UC, this was not statistically significant in the multiple regression analysis, results that are consistent with those reported in the literature [4].

Fatigue is a chronic condition affecting more than half of the patients with IBD in clinical remission and more than 80% of those with active disease. It is characterized by lack of energy and exhaustion that cannot be explained by the physical effort, does not improve with rest and leads to a limitation in daily activities [13]. The lack of energy may be a common symptom in many inflammatory conditions, patients with IBD reporting it to be more burdensome than the gastrointestinal symptoms, such as diarrhea [22]. Although common, fatigue is often underdiagnosed and overlooked by physicians [13].

Our study shows that fatigue has a detrimental effect on the HR-QoL of patients with IBD, with a strong and significant correlation between IBDQ32 score and the FACIT Fatigue score. This finding confirms data from the literature, outlining the importance of addressing this issue and increasing awareness among physicians [8,13,23,24].

Fatigue was associated with lower scores in all the domains of the IBDQ score. Moreover, it is the only factor associated with a lower score in the bowel symptoms’ domain (i.e., patients that have a lower QoL due to their bowel adverse symptoms are more affected by fatigue). Even if due to the cross-sectional design the directionality cannot be evaluated, it is reasonable to consider that bowel symptoms may lead to fatigue. Patients tend to restrict their diet because of the fear of experiencing unpleasant gastrointestinal symptoms, leading to a decrease in their QoL [21].

The patients’ diet and eating patterns play an important role in the etiology of fatigue and it has been shown that food avoidance and restrictive dietary behavior are common among patients with IBD, with prevalences up to 28–89% and 41–93%, respectively [25]. This can lead to nutritional deficiencies, which are key elements in the development of fatigue [13,26].

Of note, in our study there was a smaller proportion of patients presenting severe fatigue as defined by a FACIT Fatigue score <30, compared to results reported by a French nationwide survey (6.2% vs. 29.6%) [27].

Symptoms of anxiety and depression were present in 33% and 16.5% of patients, which is similar to data reported in other studies [9,28]. Interestingly, only anxiety had a significantly negative impact on the HR-QoL in the multivariate analysis. Even if statistically significant in the bivariate analysis, depression was not significant in the multivariate analysis. This may be explained by the lower number of patients with depression in our cohort, and even if the effect size is clinically significant (IBDQ32 Δ = 33.14), it did not reach statistical significance.

Anemia, a known factor that alters the HR-QoL in patients with IBD [29], was significant in the multivariate analysis only for the emotional function domain of the IBDQ32 score (*p* = 0.03) in our study. Because patients in this study were in clinical remission, there were only 10 patients with anemia (10.3%) and the hemoglobin levels were only slightly below normal (mean Hb levels in men with anemia: 12.7 g/dL, mean Hb levels in women with anemia: 10.9 g/dL).

Age was not significantly associated with a lower HR-QoL maybe due to the fact that the study population did not have a big variability in age, which had a median of 39 years and an IQR of 29–47 years; however, similar results were reported by other studies [27].

Regarding the level of education, the majority of patients had a medium or high level of education, with the IBDQ score being almost the same in these two categories. Patients with a lower education level also had a lower IBDQ score, which could be explained by a lower socio-economic status, but there were only few patients in this category. This could be explained by the fact that patients with lower levels of education tend to be more reluctant to participate in surveys [30,31].

Even if patients treated with ustekinumab had a lower HR-QoL, this was not statistically significant. Of note, ustekinumab is the latest biological therapy approved in Romania and could only be used as a second line treatment for patients with CD at the time this study was conducted, which can explain the lower HR-QoL of these patients.

### Strenghts and Limitations

This study brings new data regarding the factors that impair the IBD patients’ QoL, during the period of clinical remission. It offers a thorough evaluation of the HR-QoL and identifies factors that impact the patients’ HR-QoL by bowel symptoms and systemic symptoms or by affecting their emotional or social function (the domains comprised by the IBDQ32 score). In addition, patients included in this study did not have perianal disease or ostomy, which are known to have a significant negative effect on the patient’s HR-QoL [32]. Even if the luminal disease is well controlled with the treatment, patients affected by perianal disease may still need medical or surgical care for it. Therefore, this study shows that even in patients that are considered to be optimally treated, there is still much to be done to improve their QoL. The assessment of these factors with their early identification and correction could lead to an increase in the patients’ QoL.

One of the limitations of this study is its cross-sectional design, which could not offer a causal relationship between exposure and outcome. Another limitation is that the remission was determined only on clinical basis, without an endoscopic evaluation at the moment inclusion to confirm the remission, which could have drastically decreased the patient’s intent to participate in this study. In addition, being conducted in a tertiary center, the majority of patients were on biologic therapy and may have had a more severe disease.

## 5. Conclusions

The study shows that the HR-QoL is significantly impaired by fatigue and anxiety. A lower HR-QoL is also associated with the exposure to a higher number of biological treatments.

Increasing awareness of the factors that lead to an impairment in the QoL of patients with IBD and addressing them properly could result in a net improvement in their QoL.

Future research should focus on further identification of factors that alter the HR-QoL and evaluate interventions on specific factors that could be associated with an improvement in HR-QoL.

## Figures and Tables

**Table 1:** Characteristics of study population.

Characteristic	
Gender - Men, *n* (%) - Women, *n* (%)	62 (63.9%) 35 (36.1%)
Phenotype - Crohn’s disease, *n* (%) - Ulcerative colitis, *n* (%)	65 (67%) 32 (33%)
Median age (IQR)	39 years (29–47)
Current smokers, *n* (%)	60 (61.9%)
Median disease duration (IQR)	5 years (2–10)
Treatment - Biologic, *n* (%) - Conventional, *n* (%)	93 (95.9%) 4 (4.1%)
Type of biological treatment - Infliximab - Adalimumab - Vedolizumab - Ustekinumab	55 (56.7%) 10 (10.3%) 26 (26.8%) 2 (2%)
Exposure to biological treatments: Number of biological treatments - 0–1, *n* (%) - >1, *n* (%)	75 (77.3%) 22 (33.7%)
History of IBD surgery	31 (31.9%)
Level of education - Inferior, *n* (%) - Medium, *n* (%) - Superior, *n* (%)	6 (6.2%) 43 (44.3%) 48 (49.5%)
Anemia, *n* (%)	10 (10.3%)
Mean IBDQ score (+/− SD)	190.54 (+/− 22.7)
FACIT-Fatigue - Mean (+/− SD) - Severe fatigue, *n* (%) (FACIT-Fatigue < 30)	42.25 (+/− 8.2) 6 (6.2%)
Presence of symptoms of anxiety, *n* (%)	32 (33%)
Presence of symptoms of depression, *n* (%)	16 (16.5%)

**Table 2:** Analysis of factors that altered the HR-QoL.

Bivariate Analysis			Multivariate Analysis
Parameter	IBDQ32 Score	*p* Value	β coef., *p* Value
Gender	Mean in males: 194.9 Mean in females: 182.7 Δ = 12.2	0.016	β = −1.21, *p* = 0.60
Age	r = −0.002	0.97	β = 0.07, *p* = 0.43
Phenotype	Mean in CD: 186.9 Mean in UC: 197.81 Δ = 10.91	0.007	β = 2.91, *p* = 0.27
Disease duration	r = −0.24	0.017	β = −0.18, *p* = 0.43
>1 biological therapy	Mean in 0–1 biological therapy: 196.38 Mean in >1 biological therapy: 170.63 Δ = 25.75	<0.001	β = −7.25, ***p* = 0.02**
History of IBD surgery	Mean in ‘yes’ group: 186.41 Mean in ‘no’ group: 192.48 Δ = 6.07	0.24	β = 3.66, *p* = 0.15
Level of education	Mean IBDQ: - Inferior: 181.33 - Medium: 191.86 - Superior: 190.52	0.69	β = 1.39, *p* = 0.43
Anemia	Mean in anemic patients: 192.95 Mean in non-anemic patients: 169.6 Δ = 23.25	0.02	β = −0.07, *p* = 0.98
FACIT-Fatigue	r = 0.89	<0.001	β = 1.90, ***p* < 0.001**
Symptoms of anxiety	Symptoms of anxiety present: 166.93 Symptoms of anxiety absent: 202.16 Δ = 35.23	<0.001	β = −11.38, ***p* < 0.001**
Symptoms of depression	Symptoms of depression present: 162.87 Symptoms of depression absent: 196.01 Δ = 33.14	<0.001	β = 2.59, *p* = 0.49

**Table 3:** Subanalysis of factors that lower the score of each domain of IBDQ32 score.

Parameter	IBDQ B Bowel Symptoms (β coef., *p* Value)	IBDQ S Systemic Symptoms (β coef., *p* Value)	IBDQ E Emotional Function (β coef., *p* Value)	IBDQ SF Social Function (β coef., *p* Value)
Gender	β = 0.53, *p* = 0.60	β = −0.11, *p* = 0.82	β = −0.26, *p* = 0.81	β = −1.37, *p* = 0.054
Age	β = −0.02, *p* = 0.47	β = 0.06, *p* = 0.004	β = 0.09, *p* = 0.04	β = −0.04, *p* = 0.09
Phenotype	β = 1.57, *p* = 0.18	β = −0.37, *p* = 0.51	β = 0.75, *p* = 0.56	β = 0.96, *p* = 0.22
Disease duration	β = 0.01, *p* = 0.87	β = 0.05, *p* = 0.29	β = −0.21, *p* = 0.06	β = −0.04, *p* = 0.56
>1 biological therapy	β = −1.94, *p* = 0.16	β = −0.54, *p* = 0.51	β = −1.25, *p* = 0.41	β = −3.60, *p* < 0.001
History of IBD surgery	β = 1.94, *p* = 0.09	β = 0.04, *p* = 0.93	β = 2.37, *p* = 0.06	β = −0.69, *p* = 0.37
Level of education	β = 0.84, *p* = 0.28	β = −0.04, *p* = 0.90	β = 0.06, *p* = 0.94	β = 0.52, *p* = 0.33
Anemia	β = 3.08, *p* = 0.06	β = 0.47, *p* = 0.55	β = −3.75, *p* = 0.03	β = 0.12, *p* = 0.91
FACIT-Fatigue	β = 0.40, *p* < 0.001	β = 0.38, *p* < 0.001	β = 0.80, *p* < 0.001	β = 0.31, *p* < 0.001
Symptoms of anxiety	β = −1.80, *p* = 0.19	β = −1.67, *p* = 0.01	β = −8.25, *p* < 0.001	β = 0.34, *p* = 0.71
Symptoms of depression	β = 1.60, *p* = 0.33	β = 0.28, *p* = 0.72	β = −0.98, *p* = 0.59	β = 1.68, *p* = 0.14

## Data Availability

The data that support the findings of this study are available from the corresponding author, upon reasonable request.
